# Generation of immature retinal neurons from proliferating cells in the pars plana after retinal histogenesis in mice with retinal degeneration

**Published:** 2009-01-23

**Authors:** Koji M. Nishiguchi, Hiroki Kaneko, Makoto Nakamura, Shu Kachi, Hiroko Terasaki

**Affiliations:** Nagoya University Graduate School of Medicine, Department of Ophthalmology, Showa-ku, Japan

## Abstract

**Purpose:**

To study the differentiation of immature retinal neurons/retinal precursors in the ciliary epithelium after retinal histogenesis in mice with inherited or acquired retinal degeneration.

**Methods:**

Immunoreactivity to anti-recoverin, rhodopsin, and Pax6 antibodies and binding to peanut agglutinin were analyzed histologically. The distribution and differentiation of immature retinal neurons/retinal precursors in the ciliary epithelium of mice with inherited (C3H/HeJ) and acquired (C57BL mice injected with 60 mg/kg N-methyl-N-nitrosourea) retinal degeneration were assessed. Proliferating retinal progenitors were labeled with bromodeoxyuridine (BrdU), and they were studied histologically using retinal markers.

**Results:**

Many cells of rod and cone photoreceptor lineage were identified within the ciliary epithelium of the pars plana in adult mice with inherited retinal degeneration. Tracking experiments using BrdU indicated that some of recoverin-positive cells in the pars plana (approximately 3%) were generated after retinal histogenesis, and few were produced at or after postnatal day 24 (P24). The induction of acquired retinal degeneration in adult wild-type mice (P30) increased the number of BrdU-positve cells by roughly fourfold and recoverin-positive cells by approximately 17-fold in the pars plana. Moreover, some (approximately 1.5%) of the recoverin-positive cells were newly generated from dividing retinal progenitors in the adult pars plana.

**Conclusions:**

In response to retinal damage, an increased number of immature retinal neurons/retinal precursors was observed in the pars plana of mice with acquired and inherited retinal degeneration. Some of these cells differentiated from proliferating cells even after retinal histogenesis.

## Introduction

In fishes and amphibians, a circumferential zone in the retinal margin, called the ciliary marginal zone (CMZ), produces all types of retinal neurons, and thereby continuously regenerates the retina throughout life [1-3]. While the CMZ increases its production of new neurons in response to retinal injury, neurogenesis by the intrinsic stem cells within the mature retina in fishes or neural transdifferentiation of the retinal pigment epithelium in most amphibians can also take place to promote the regeneration of the retina [1-3]. However, in mammals, despite the identification of adult germinal zones for neural regeneration of areas in the central nervous system [4,5], such as the subgranular zone for the olfactory bulb [6,7] and the utricular sensory epithelium for the inner ear [8], a zone for retinal regeneration capable of replacing lost neurons has not been defined. There is evidence suggesting that the adult ciliary epithelium contains stem cells that have the potential to proliferate and express markers specific to retinal neurons in mammals [9-14], including humans [15]. However, the in situ distributions or roles of these cells are still unclear. Recently, we found that the neuroblast layer, a retinal layer composed of retinal progenitor cells seen only during retinal development, extended into the ciliary epithelium of the pars plana during ocular development in wild-type mice [16]. In addition, many cone and rod photoreceptor precursors (termed immature photoreceptors/photoreceptor precursors in the current study) in various stages of morphological differentiation were identified in the pars plana during retinal histogenesis. However, once the gross development of the retina was complete, such precursors were rare in the ciliary epithelium. Nonetheless, these observations suggested that the ciliary epithelium of the pars plana may play an important role in the generation of retinal neurons.

In the brain, it is well known that neural stem cells proliferate in response to neural damage even in adult mammals [17-19]. Consistent with these reports, we have also reported an increased number of cells immunopositive for a retinal marker in the adult pars plana after toxin-induced retinal degeneration [16]. However, it is not known whether these cells were generated from dividing cells or differentiated from post-mitotic cells. In this study, we examined two mouse models with retinal degeneration, inherited and acquired, and found that a small fraction of immature retinal neurons/retinal precursors was newly generated from dividing cells after retinal histogenesis.

## Methods

### Animals

All experimental procedures were performed in accordance with the guidelines of the Institute for Laboratory Animal Research (Guide for the Care and Use of Laboratory Animals) and Nagoya University School of Medicine for the use of animals. C57BL/6J mice were used as wild-type controls and for the N-methyl-N-nitrosourea (MNU; Clea, Tokyo, Japan) injection study. C3H/HeJ mice (*rd1* mice; Clea, Tokyo, Japan) were used as a murine model of inherited retinal degeneration [20]. All mice were fed on a basal diet (CE-2; Clea, Tokyo, Japan) and water. They were kept on a 12-h light-dark cycle in a standard cage placed in a temperature- and humidity-controlled environment. The number of mice used in each quantitative experiment is indicated in Table 1. A total estimate of at least 200 mice was used throughout the study (128 mice were used for quantification purpose).

**Table 1:** Summary of histograms presented in Figure 3, Figure 6, Figure 7, and Figure 8.

**Figure**	**Group**	**Mean±SEM**	**Unit**	N*
Figure 3C	Rho	27.7±2.6	%	6
	PNA	60.9±5.1	%	6
	Rcv	11.3±4.6	%	6
Figure 3F	rd1 P60	11.3±0.6	Cells	8
	rd1 P120	2.7±0.3	Cells	6
	Wt P60	0.3±0.1	Cells	6
Figure 6E	P6	7.5±0.8	%	8
	P12	2.9±0.4	%	8
	P18	0.5±0.2	%	6
	P24	0.1±0.1	%	8
	Wt P6	0.5±0.5	%	8
	Wt P12	0.0±0.0	%	8
Figure 6F	P9	1.3±0.4	%	6
	P12	5.7±0.7	%	7
	P20	11.8±1.2	%	7
	P30	7.5±0.8	%	8
Figure 7C	3h	1.3±0.6	%	8
	11h	6.0±1.8	%	8
Figure 8F	MNU+	5.5±1.2	Cells	8
	MNU-	1.2±0.3	Cells	12
Figure 8G	MNU+	16.1±2.0	Cells	8
	MNU-	0.9±0.2	Cells	12
Figure 8H	Rho	7.5±2.4	%	8
	PNA	77.3±4.2	%	8
	Rcv	15.1±3.8	%	8
Figure 8I	MNU+	1.5±0.7	%	8
	MNU-	0.0±0.0	%	12

The numeric data (mean±SEM) for each histograms indicated by the Figure and group names are presented along with unit and the number of animals used for quantification.

### BrdU labeling

To label cells in the S-phase of the cell cycle, we injected mice intraperitoneally with bromodeoxyuridine (BrdU; Sigma, St. Louis, MO). A dose of BrdU used for each experiment is indicated in the figure legend. These mice were euthanized either by inhalation of 100% CO_2_ or by cervical dislocation after 2 h to 24 days to collect the eyes.

### Immunohistochemistry

Immunohistochemical analyses were performed as described previously [16,21]. Sections and flat-mount specimens were stained with primary antibodies for BrdU (1:1,000; Oxford Biotechnology, Oxford, UK), Pax6 (1:200; marker for retinal progenitors; Developmental Studies Hybridoma Bank, Iowa City, IA), rhodopsin (1:1,000; marker for rods; Chemicon, Temecula, CA), recoverin (1:1,000; marker for rods and cones and subset of cone bipolar cells; Chemicon), synaptophysin (1:1,000; marker for synaptic vesicles; Sigma) and K_i_-67 (1:1,000; cell proliferation marker [22]; BD PharMingen; San Diego, CA) followed by combinations of 1:1,000 Alexa 405-, 1:1,000 488-, and 568-conjugated secondary antibodies (1:1,000; Molecular Probes, Eugene, OR), PNA (1:100; marker for the inner/outer segments and pedicles of cones [23,24]; Molecular Probes), and diamidino-2-phenylindole (1:1,000; DAPI; Molecular Probes). After the staining procedure, mild overnight pressure was applied to the flat-mount specimens to remove the undesired retinal/choroidal/scleral folds. As a result, the thickness of the retina scanned for histological analyses would not necessary correspond to that in living eyes.

### Identification of cells in the pars plana within flat-mounts

**Figure 1:**
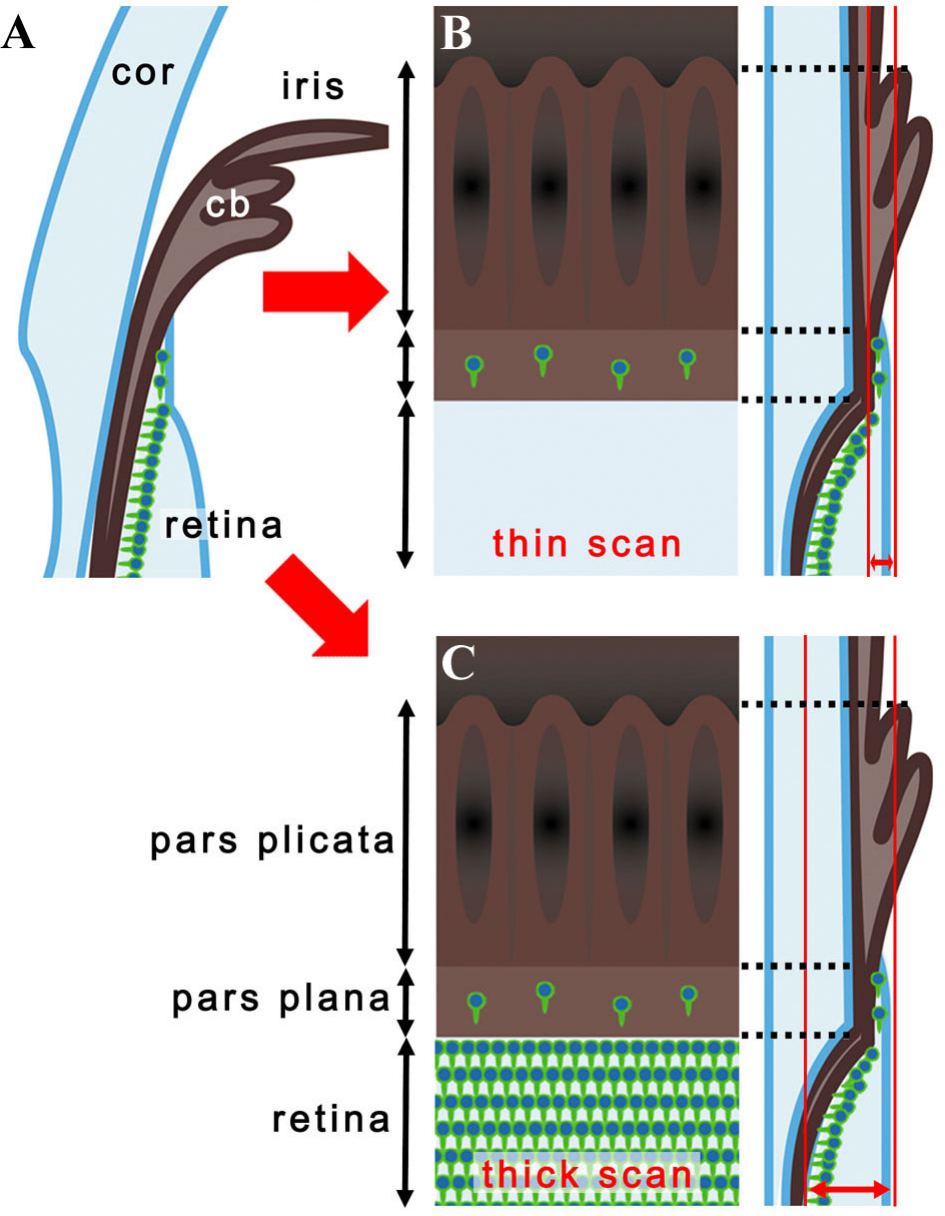
Cilioretinal flat-mounts. **A:** The diagram illustrates the histological section of the retina and ciliary body. Photoreceptors and their precursors are shown as green cells in the deep retina and pars plana. **B:** Thin scans of the cilioretinal flat-mounts focused at the level of the ciliary epithelium showed cells positive for photoreceptor markers in the pars plana as sporadic cells aligned circumferentially along the retinal margin. With this scanning method, no photoreceptors could be seen in the retina. **C:** In thick scans of flat-mount specimens, dense photoreceptors in the deep retina were also visualized showing a sharp demarcation at the cilioretinal border. Abbreviations: cornea (cor); ciliary body (cb).

With regard to the images of flat-mount specimens acquired by confocal microscopy (Eclipse C-1/ Nikon, Tokyo, Japan) cells in the pars plana were presented in two different ways: thin scan and thick scan (Figure 1). The former is a scan with its focus on the ciliary epithelium and the retinal surface (Figure 1B). When evaluating photoreceptor markers, we visualized only cells in the pars plana in a circumferential and sparse distribution. The latter was presented as an image of a single scan or as a merged image of 2 to 4 scans (the detail is indicated in the corresponding legend) that included the cells of ciliary epithelium as well as those of the photoreceptor layer (Figure 1C). Only some images were presented with thin scan (indicated in the legend) while all the other flat-mount data were presented with thick scan. Using this method, we found cells in the pars plana showed a distribution similar to that of the thin scan. Meanwhile, cells in the retina typically showed much denser staining demarcating the cilioretinal border. In addition, the following features distinguished the ciliary body from the retina. First, the inner surface of the pars plana was flat, showing a contour similar to the retina, while in the deeper layers it often had discernable folds that appeared to be the continuum of the pars plicata. Second, an intervening space or gap was frequently detected between immunopositive cells at the retinal margin and pars plana.

Almost all figure panels that were obtained with thick scans are merged images of multiple scans as described in the legend. The interval of each optical section comprising the merged image was set at approximately 75% of the thickness of the scan itself. This is to merely show the retina as a “plane” with dense photoreceptors. A single thick scan was applied to all images obtained for statistical purpose (this is enough to just visualize the cilioretinal border and the cells in the pars plana). A thick scan was adopted for statistical purposes because a thin scan had a higher chance of underestimating the cells in the pars plana, which might result in larger inter-sample variations. In addition, it was more convenient to visualize the cilioretinal border by thick scan in the eyes with a thin degenerated retina. In wild-type mice or developing *rd1* mice that have thick retinas, we often found it easier to isolate cells in the pars plana with a thin scan.

### MNU injection

C57BL/6J mice were injected intraperitoneally with 60 mg/kg MNU (Sigma). Seven days after MNU administration, severe photoreceptor degeneration was observed in MNU-injected animals [25].

### Quantification and statistical analysis

The numbers of cells positive for recoverin, rhodopsin, PNA, BrdU, and K_i_-67 per 320 µm width of the pars plana were quantified from at least 3 independent images obtained per animal. The number of animal used for each experiment is outlined in Table 1. For quantification of cells in the pars plana, images were obtained with a single scan with its focus on the ciliary epithelium. Cells that encompassed both the retina and pars plana were excluded from the analyses. Student’s unpaired *t*-test was used for the statistical analysis of the differences. p<0.05 was considered significant.

## Results

### Pars plana of *rd1* mice during ocular development

**Figure 2:**
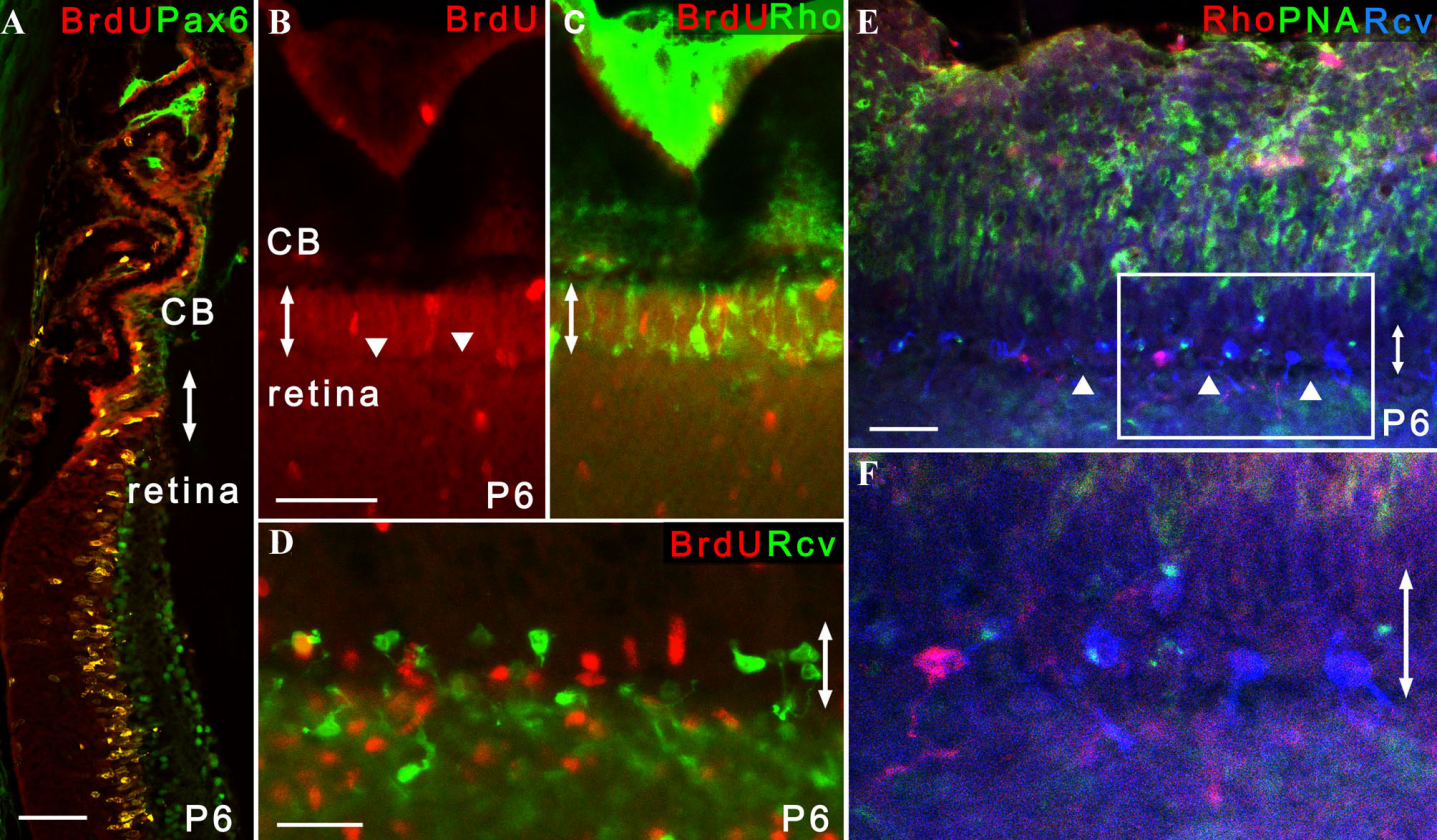
Identification of photoreceptor lineage cells in the pars plana in P6 *rd1* mice. Double-headed arrows indicate the pars plana. All the images were obtained from P6 *rd1* mice. **A:** Cells positive for both BrdU and Pax6 formed the neuroblast layer that extended both in the peripheral retina and ciliary body (n=6). **B, C:** The neuroblast layer composed of BrdU-positive cells extended into the pars plana in which rhodopsin-positive cells were identified (n=13). Note that a small linear gap (arrowhead) demarcating the pars plana and retina can be seen in **B**. **C** is a merged image of **B** and the result of rhodopsin staining. **D:** Recoverin-positive cells were observed in sparse distribution in the pars plana, while the peripheral retina showed dense recoverin immunoreactivity (n=12). **E, F:** The majority of recoverin-positive cells in the pars plana were cells of cone photoreceptor lineage with a short PNA-positive process (n=7). **F** is an enlarged image of **E**. Note that a linear gap (arrowhead) demarcating the pars plana and retina can be seen. **B, C, E**, and **F** are presented as a thin scan (a single scan 7.1 μm thick). **D** is a thick scan merged from 2 images (each scan was 10.0 μm thick). A dose of BrdU injected was 150 mg/kg. Scale bar equals 25 μm in **D** and **F** and 50 μm in **A** and **B**. Abbreviations: rhodopsin (Rho); recoverin (Rcv).

To avoid ambiguous interpretation, we defined retinal progenitors as proliferating cells with the potential to generate retinal neurons. Post-mitotic cells that had some morphological features of neurons in the ciliary body were defined as immature retinal neurons/retinal precursors (termed retinal precursors in the previous report) [16] in this study. First, we examined the hypothesis that retinal progenitors or immature retinal neurons/retinal precursors are also present in the developing ciliary epithelium of *rd1* mice. In *rd1* mice, photoreceptors begin to degenerate rapidly at postnatal day 12 (P12), shortly after histogenesis of the retina is complete [20]. Mitotic cells were labeled by intraperitoneal injection of BrdU into P6 *rd1* mice 2 h before enucleation. Histological sections showed that cells positive for both BrdU and Pax6 were most abundant in the peripheral retina and ciliary epithelium, many of which formed the neuroblast layer (Figure 2A) similar to the observation in wild-type mice [16]. In flat-mounts, the BrdU-positive cells comprising neuroblast layer in the retina extended into areas of the pars plana where recoverin-positive or rhodopsin-positive cells were also found (Figure 2B-D). Further analyses of the pars plana revealed that most of the recoverin-positive cells were of cone photoreceptor lineage positive for PNA (Figure 2E,F). Such cells had various degrees of morphological features of immature cone photoreceptors or its precursors in the developing retina. These include a large budding at the end of one of its processes called the pedicles, which are sometimes as large as the cell body, and a conical process at the end of the other process corresponding to the inner and outer segments. Unfortunately, calculating the proportion of rod versus cone lineage cells in the pars plana was difficult because of the relatively weak signal and the high background of PNA staining, particularly during retinal development [23,26]. Nonetheless, these finding were consistent with the results of wild-type mice that showed the development of the pars plana and peripheral retina were closely related to each other until the latest stage of postnatal ocular development [16].

### Pars plana of adult rd1 mice

**Figure 3:**
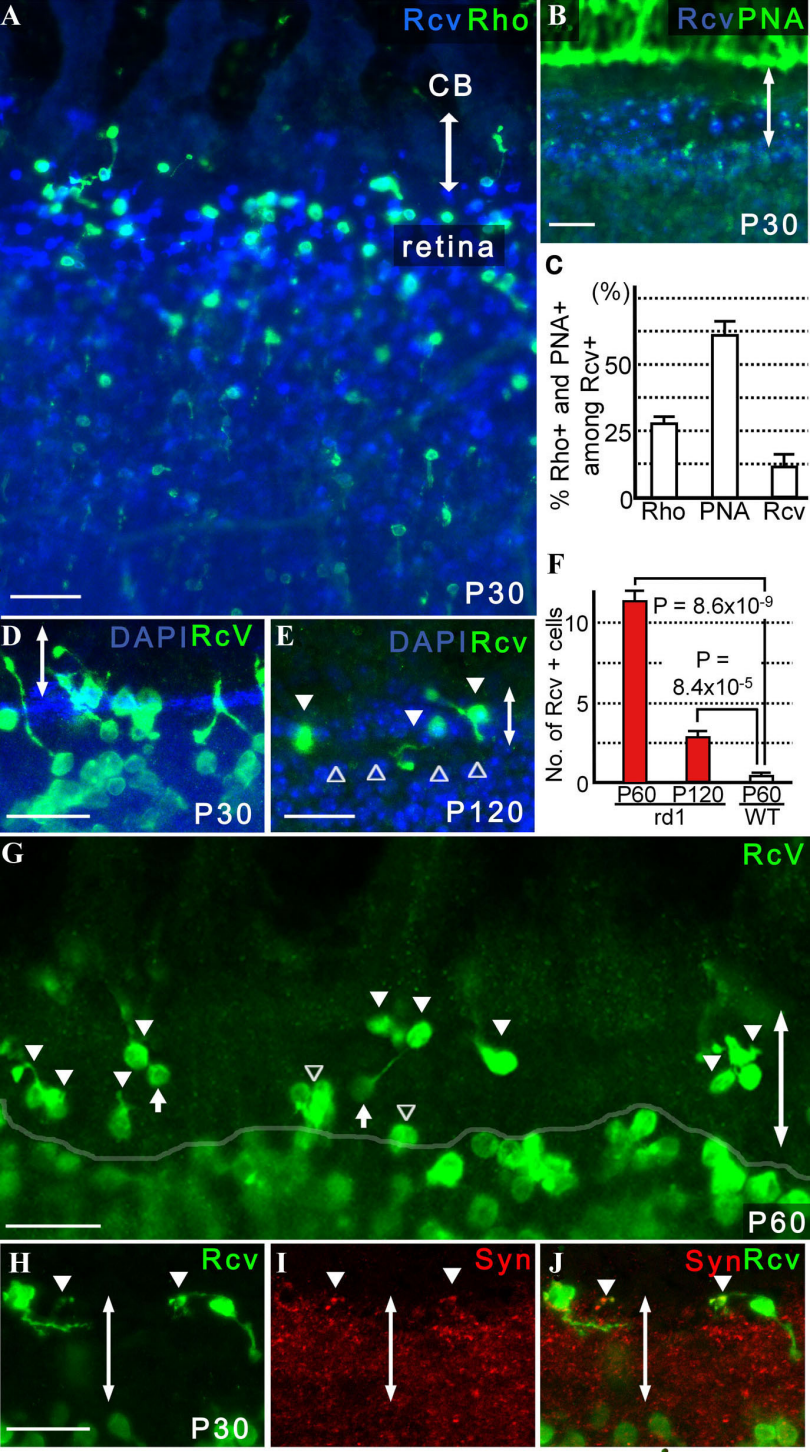
Retinal precursors were identified in the pars plana of adult *rd1* mice. Double-headed arrows indicate the pars plana. **A**: At P30, many cells positive for rhodopsin as well as recoverin were seen in the pars plana of *rd1* mice (n=39). **B**: The majority of the recoverin-positive cells were stained with PNA (n=12). **C:** The proportions of recoverin-positive cells in the pars plana at P30 that are positive for PNA or rhodopsin (mean±SEM) are presented. After the number of recoverin-positive cells within 320 µm width of the pars plan were determined from a single optical scan (10.0 μm thick), the number of those also positive for rhodopsin and PNA were determined from the same image. Three independent images randomly obtained from the same eye were analyzed to calculate the proportion of recoverin-positive cells that were also positive for rhodopsin (Rho) or PNA (PNA) or neither (Rcv) per animal. Average proportion (%) of cells for each category was determined from 6 animals. **D:** Many retinal precursors bridged both the retina and pars plana. Note that the cilioretinal border was sharply demarcated with DAPI staining (n=24). **E:** Recoverin-positive cells (arrowhead) in the pars plana at P120. Note that a gap between the pars plana and retina (open arrowhead) was seen with DAPI staining. **F:** Increased numbers of recoverin-positive cells were observed in the pars plana of P60 (31.2 fold; n=8) and P120 (7.6 fold; n=6) *rd1* mice compared with P60 wild-type mice (n=6). The number of recoverin-positive cells within 320 µm width of the pars plan from a single optical scan (10.0 μm thick) were determined from three independent images randomly obtained from the same eye. Average number of recoverin-positive cells within 320 µm width of the pars plan (mean±SEM) were determined for each group (i.e., P60 wild-type mice, P60 rd1 mice, and P120 rd1 mice). Note that the number of recoverin-positive cells decreased with aging, from P60 to P120, in *rd1* mice. **G:** A representative image of a single thick scan used for quantification of recoverin-positive (green) cells in the pars plana (filled arrowhead) is shown. A semitransparent white line outlines the cilioretinal border. Those that encompassed the border (open arrowhead) were excluded. Note that the immunopositive cells in the deep retina are defocused, while those in the ciliary epithelium of the pars plana appear sharper. The oval structures connected to a cell body through a thin process (filled arrow) were considered cone pedicles, thus excluded from the cell count. **H, I, J:** Recoverin-positive cells in the pars plana with synaptophysin-positive synaptic vesicles are presented (**J**; n=4; arrowhead). A merged image of **H** and **I** is shown. Scale bar equals 25 μm in **D, E, G,** and **H** and 50 μm in **A** and **B**. **A, B, D, H, I**, and **J** are thick scans (merged from 2 scans; each scan was 7.1 μm thick for **A** and **B** and 3.9 μm thick for **D, G, H**, and **I,** respectively). **E** is presented as a thin scan (a single scan 10.0 μm thick). Abbreviations: rhodopsin (Rho); recoverin (Rcv); ciliary body (CB); synaptophysin (Syn).

In wild-type mice, the existence of immature retinal neurons/retinal precursors generated in the ciliary epithelium of the pars plana is mostly restricted to a short period during retinal development and is rare at P12 [16] when gross retinal histogenesis is complete [27-29]. To determine whether immature photoreceptors/photoreceptor precursors were present after retinal histogenesis in the eyes of *rd1* mice, we examined the ciliary epithelium and peripheral retina of adult *rd1* mice. Although BrdU-positive cells were generally increased in *rd1* mice compared to the wild-type controls, such cells mostly disappeared from the retina by P12 and continued to decline in number also in the ciliary epithelium (data not shown). However, many cells of rod and cone photoreceptor lineage, positive for rhodopsin and PNA respectively, were found in the pars plana of *rd1* mice at P30 (Figures 3A,B). The former comprised approximately 28% and the latter approximately 61% of recoverin-positive cells, while the identity of an estimated 11% remained unclear (Figure 3C). We found no recoverin-positive cells that were positive for both PNA and rhodopsin. Some of these immature retinal neurons/retinal precursors bridged both the ciliary epithelium and retina (Figure 3D). Rhodopsin-positive cells persisted in the pars plana up to P90 (data not shown) and recoverin-positive cells up to at least P120 (Figure 3E). Consequently, in comparison to the number of recoverin-positive cells in the pars plana of wild-type mice at P60, those of *rd1* mice at P60 and P120 were 31.2 and 7.6 fold higher, respectively (Figure 3F,G). Many of these cells had detectable synaptophysin-positive vesicles in their synaptic terminals (Figures 3H-I), suggesting that they may be capable of generating and releasing neurotransmitters. Interestingly, we also found a few recoverin-positive cells in the pars plicata of adult *rd1* mice (data not shown). Similar cells were found in the developing but not in the adult pars plicata of wild-type mice (data not shown).

**Figure 4:**
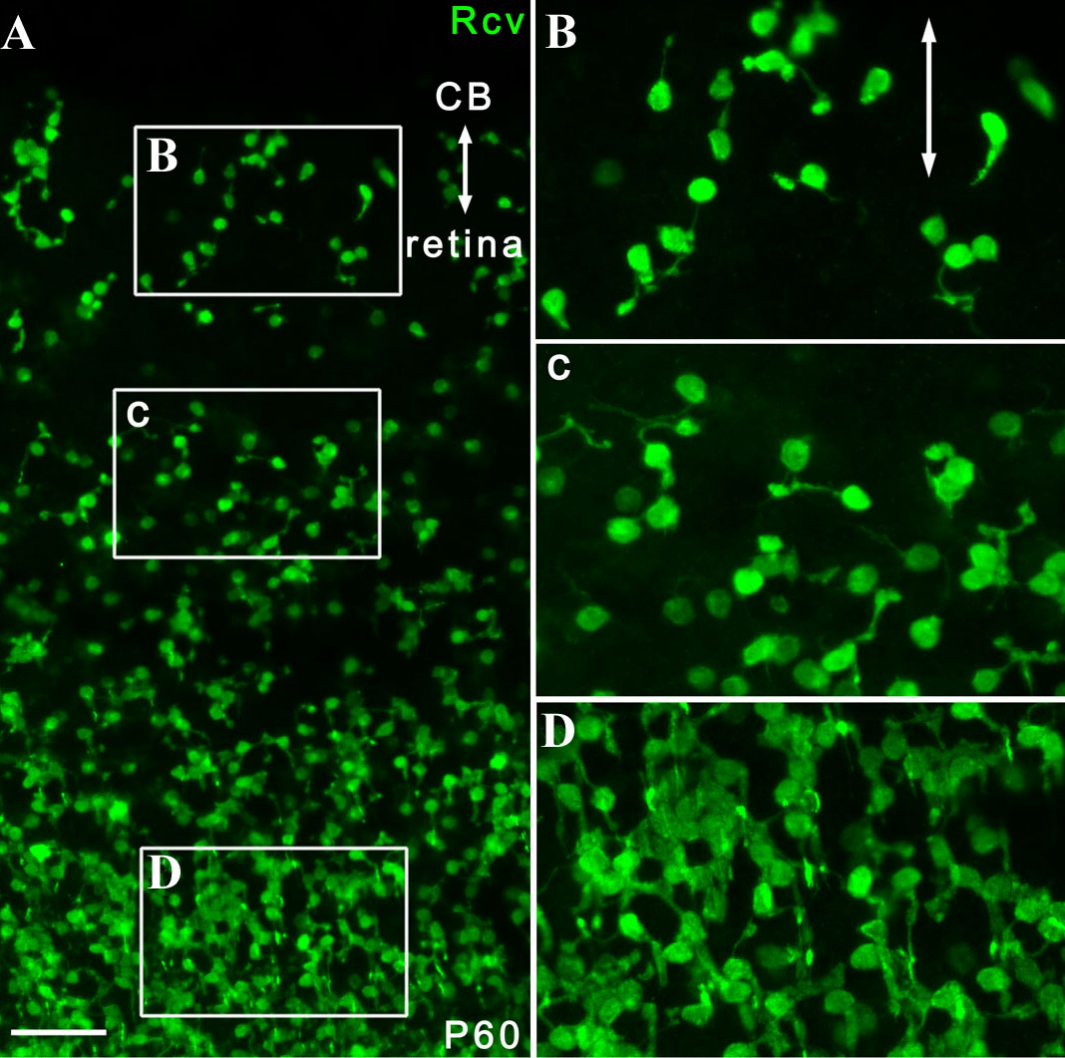
Shared morphological features of recoverin-positive cells in the pars plana and the peripheral retina in *rd1* mice. Double-headed arrows indicate the pars plana. **A:** At P60, many recoverin-positive cells in both the ciliary epithelium and peripheral retina were oriented randomly, while most of those in the posterior parts of the retina were aligned radially (n=14) in *rd1* mice. **B-D:** Selected areas from **A** are magnified. A was a thick scan (merged from 4 scans; each scan was 10.0 μm thick; see Methods for details). Scale bar equals 50 μm. Abbreviations: ciliary body (CB); recoverin (Rcv).

**Figure 5:**
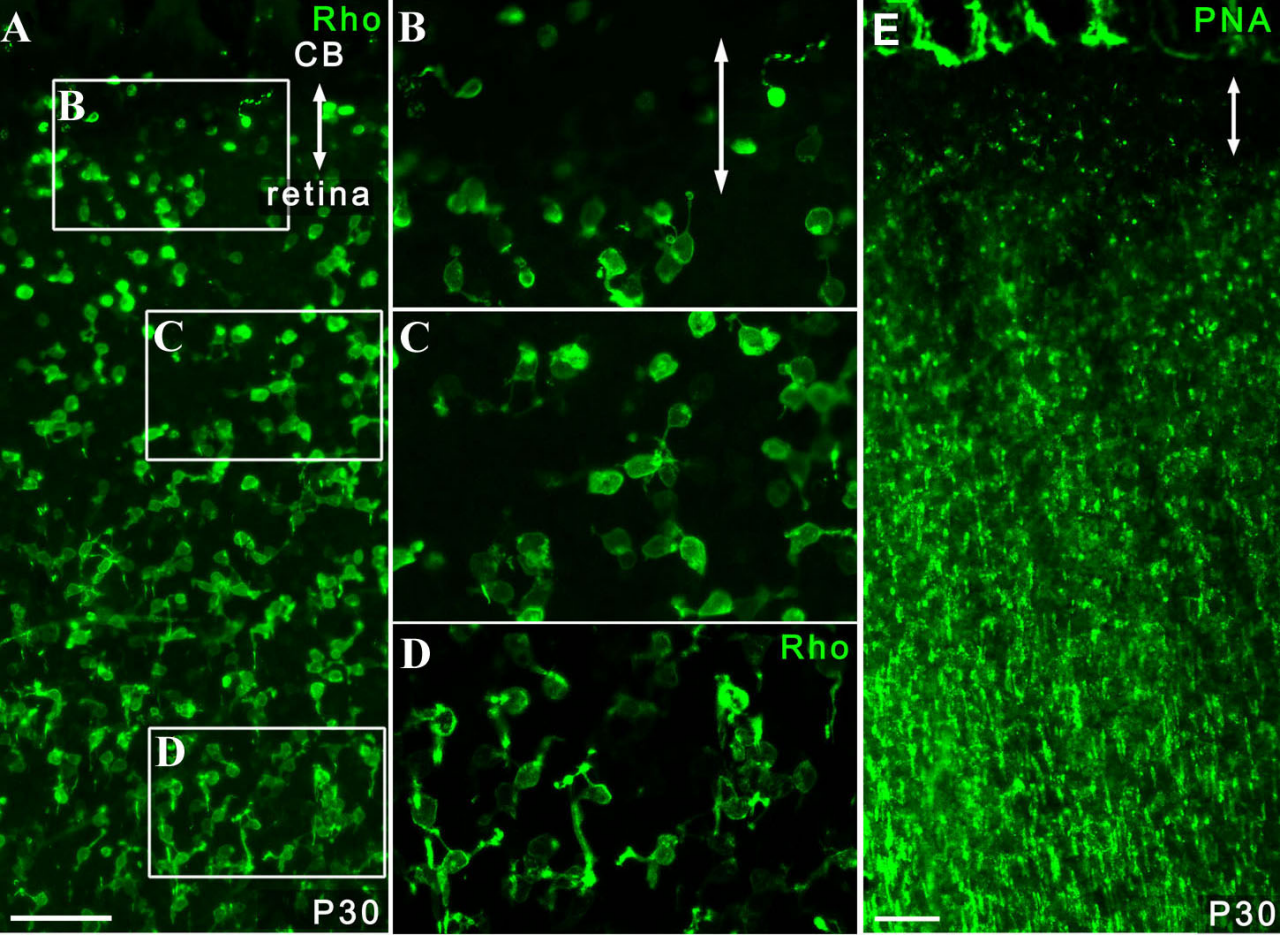
Shared morphological features of rhodopsin-positive or PNA-positive cells in the pars plana and peripheral retina in *rd1* mice. Double-headed arrows indicate the pars plana. **A:** At P30, many rhodopsin-positive cells in both the ciliary epithelium and peripheral retina were oriented randomly, while most of those in the posterior parts of the retina were aligned radially in *rd1* mice. **B-D:** Selected areas from **A** are magnified. **E:** At P30, PNA-positive processes were generally shorter in the peripheral retina and par plana compared to those in the posterior parts of the retina with longer processes in *rd1* mice. **A** and **E** are thick scans (merged from 4 scans; each scan was 10.0 μm thick). Scale bar equals 50 μm. Abbreviations: ciliary body (CB); recoverin (Rcv).

In the pars plana and areas of the peripheral retina of P60 *rd1* mice, the recoverin-positive cells showed common morphological characteristics that were distinct from the posterior parts of the retina (Figure 4). These cells had short as well as randomly oriented processes in the pars plana and peripheral retina, while those in the remaining posterior part of the retina had longer processes and were aligned radially in an organized manner. However, the location of the border of such a transition in cellular alignment and morphology varied considerably between mice and even within the same retina. Similar regional differences in cell morphology were also observed in rhodopsin- and PNA-positive cells at P30 (Figure 5).

### Generation of recoverin-positive cells after retinal histogenesis

**Figure 6:**
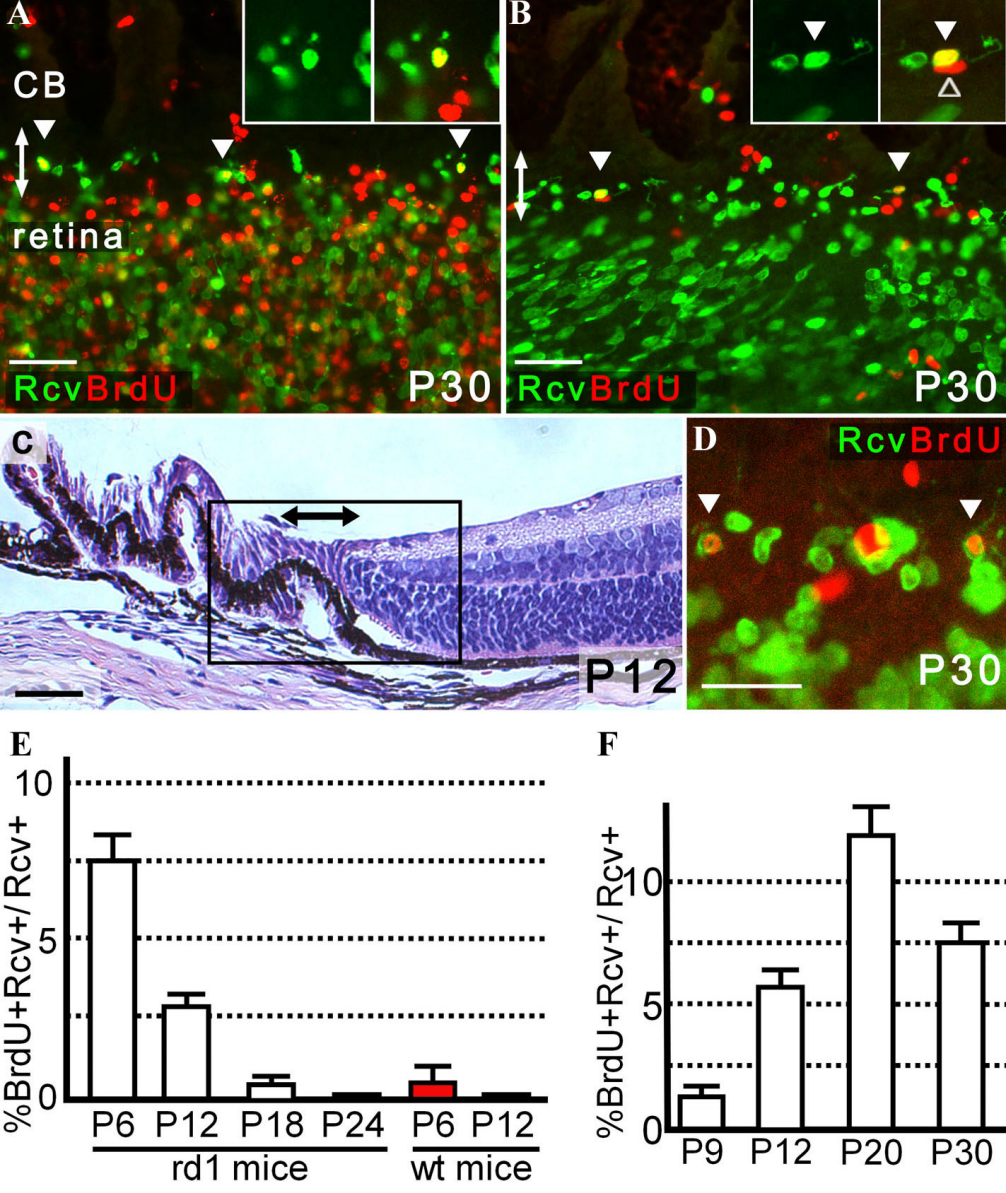
Generation of recoverin-positive cells in the pars plana after retinal histogenesis in *rd1* mice. Double-headed arrows indicate the pars plana. **A:** Cells positive for both BrdU (labeled at P6) and recoverin (arrowhead) were found in the P30 pars plana. Note that numerous cells in the neuroblast layer were labeled with BrdU at P6. **B:** Cells positive for both BrdU (labeled at P12) and recoverin (filled arrowhead) were identified in the pars plana at P30. A cell positive for BrdU but negative for recoverin is also present in the inset (open arrowhead). Only rare BrdU-positive cells, mostly blood cells (cells in the lower right corner), were identified in the retina, suggesting that gross retinal histogenesis was already complete by P12. Note that immunopositive cells in the peripheral retina showed an oblique alignment, which contrasted with those in the pars plana; the differential susceptibility of the retina and ciliary epithelium to mounting artifacts is one of the features that sometimes distinguished the cilioretinal border. **C:** Hematoxylin and eosin staining of eye section is from a P12 *rd1* mouse. The box roughly corresponds to the area from which images **A** and **B** were obtained. **D:** Cells positive for both BrdU (labeled at P18) and recoverin (arrowhead) were identified in the P30 pars plana. **E,F:** The proportion (%) of cells positive for BrdU (injected at P6, P12, P18, and P24) among recoverin-positive cells in the P30 pars plana (**E**) and similarly in the pars plana of mice (enucleated at P9, P12, P20, and P30) after BrdU injection at P6 (**F**) are presented. After the number of recoverin-positive cells within 320-µm width of the pars plan were determined from a single optical scan (10.0 μm thick), the number of those also positive for BrdU were determined from the same image. Three independent images randomly obtained from the same eye were analyzed to calculate the proportion of BrdU-positive cells among recoverin-positive cells per animal. Average proportion (%; mean±SEM) for each category were determined from the following number of animals. The numbers of mice used in each experiment is summarized in Table 1. A dose of BrdU injected was 50 mg/kg. **A** and **B** are thick scans each merged from 3 scans (each scan was 10.0 μm thick) while **D** is also a thick scan but merged image from 2 scans (each scan was 3.9 μm thick). Scale bar equals 25 μm (**D**) and 50 μm in **A, B**, and **C**. Abbreviation: recoverin (Rcv).

We identified many immature neurons/neural precursors mostly of photoreceptor lineage in the pars plana of adult as well as young *rd1* mice. However, whether these cells in the adult pars plana were produced as a result of neurogenesis, i.e., differentiation from their proliferating progenitors, after retinal histogenesis or were merely persistence of or differentiation from post-mitotic cells that were produced during ocular development remains unknown. To clarify this issue, we performed pulse-chase assays to define the temporal origins of these cells. BrdU used to label dividing cells may have negative influence on the neural differentiation [30]. To avoid such an undesired effect, a minimal dosage of BrdU injection that allowed a reliable detection of the agent was sought. We found that a BrdU dosage of 50 mg/kg was sufficient for this study after trying various injected doses of BrdU (12.5, 25, 50, 100, and 150 mg/kg) and histologically evaluating its incorporation (data not shown). Using this dosage, dividing cells were labeled with BrdU at P6, P12, P18, and P24, and the fates of these cells were examined later at P30. Anti-recoverin antibody that mostly stained cells of photoreceptor lineage provided the best staining quality with least background was used for the neural marker. Numerous cells were labeled with BrdU injection at P6, i.e., during retinal histogenesis. When these mice were later examined at P30, some recoverin-positive cells with morphological features of retinal neurons that incorporated BrdU were found in the ciliary epithelium (Figure 6A). These cells were evaluated thoroughly by continuous confocal scanning microscopy to confirm that the BrdU-positive nucleus resided within the recoverin-positive cells. Among the recoverin-positive cells in the pars plana, approximately 7.5% were BrdU-positive and were generated after P6 (Figure 6E). By P12, histogenesis of the retina was complete [20] as evidenced by examination of histological sections (Figure 6C) and the presence of rare BrdU-positive cells only in the peripheral retina (Figure 6B). This is also consistent with our previous quantitative evaluation of BrdU positive cells in retinal sections from developing mice [16]. Analysis of mice injected with BrdU at P12 indicated that an estimated 2.9% of recoverin-positive cells in the pars plana were generated after histogenesis, i.e., P12 (Figures 6B,E). The percentage declined further in the eyes with BrdU labeling at P18 (Figures 6D,E). The evidence of neurogenesis was extremely rare when mice were injected with BrdU at P24. Similar experiments using wild-type mice indicated that the evidence of neurogenesis in the P30 pars plana observed when BrdU was injected at P6 became undetectable with injection at P12 (Figure 6E).

**Figure 7:**
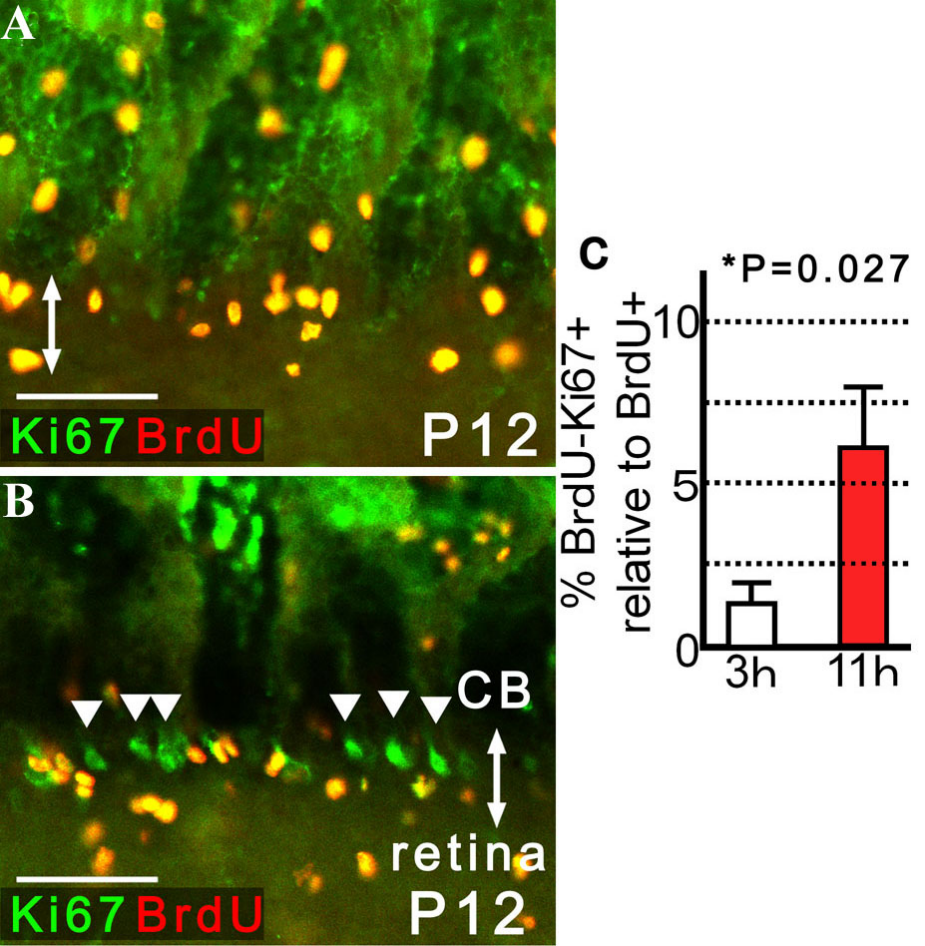
Presence of proliferating cells in the pars plana that escaped BrdU labeling and detection. The eyes were enucleated 3 or 11 h after intraperitoneal injection of BrdU (50 mg/kg) into P12 *rd1* mice. Cilioretinal flat-mounts were stained with antibodies against BrdU and K_i_-67, both of which are markers for cells proliferation. Double-headed arrows indicate the pars plana. **A:** At 3 h after BrdU injection, the cells positive for BrdU incorporation grossly matched those immunopositive for K_i_-67 in the pars plana. **B:** By 11 h after BrdU injection, clusters of cells positive for K_i_-67, but negative for BrdU (arrowheads), aligned circumferentially in selected areas of the pars plana. No such cluster was seen in the pars plicata or retina. **A** and **B** are presented as a thin scan (a single scan 10.0 μm thick). **C**: The proportion (%) of cells negative for BrdU but positive for K_i_-67 (BrdU-K_i_-67+) relative to BrdU-positive cells (BrdU+) are presented to evaluate the presence of mitotic cells that escaped BrdU labeling or detection. An increased percentage of BrdU-K_i_-67+ cells was found in the pars plana 11 h after BrdU injection compared to 3 h after (4.60-fold; means±SEM). After the number of BrdU-positive cells within 320 µm width of the pars plana were determined from a single optical scan (10.0 μm thick), the number of those immunopositive for K_i_-67, but negative for BrdU, were determined from the same image. Three independent images randomly obtained from the same eye were analyzed to calculate the relative proportion of K_i_-67-positive and BrdU-negative cells versus BrdU-positive cells per animal. Average proportion (%; mean±SEM) were determined from 8 animals each for P12 *rd1* mice sacrificed 3 and 11 h after BrdU injection. Scale bar equals 50 μm.

Next, we injected BrdU at P6 and analyzed the number of recoverin-positive cells with and without BrdU incorporation in the pars plana of *rd1* mice at P9, P12, P20, and P30. The evidence of neurogenesis was detectable by P9, 3 days after BrdU injection. The rate of cells positive for BrdU among recoverin-positive cells in the pars plana became greatest at P20, 14 days after BrdU injection, but showed a modest decrease at P30 (Figure 6F). The number of newly generated immature neurons/neural precursors after histogenesis may actually be greater than detected in the present study, as the presence of unlabeled proliferating cells that escaped the single injection of BrdU was confirmed (Figure 7); these unlabeled cells might also give rise to immature retinal neurons/retinal precursors. Interestingly, such cells negative for BrdU and positive for K_i_-67 were mostly observed in the pars plana and occasionally formed a cluster that resembled the circumferential distribution of retinal precursors (Figure 7B).

Neurogenesis in mice with acquired retinal degeneration

**Figure 8:**
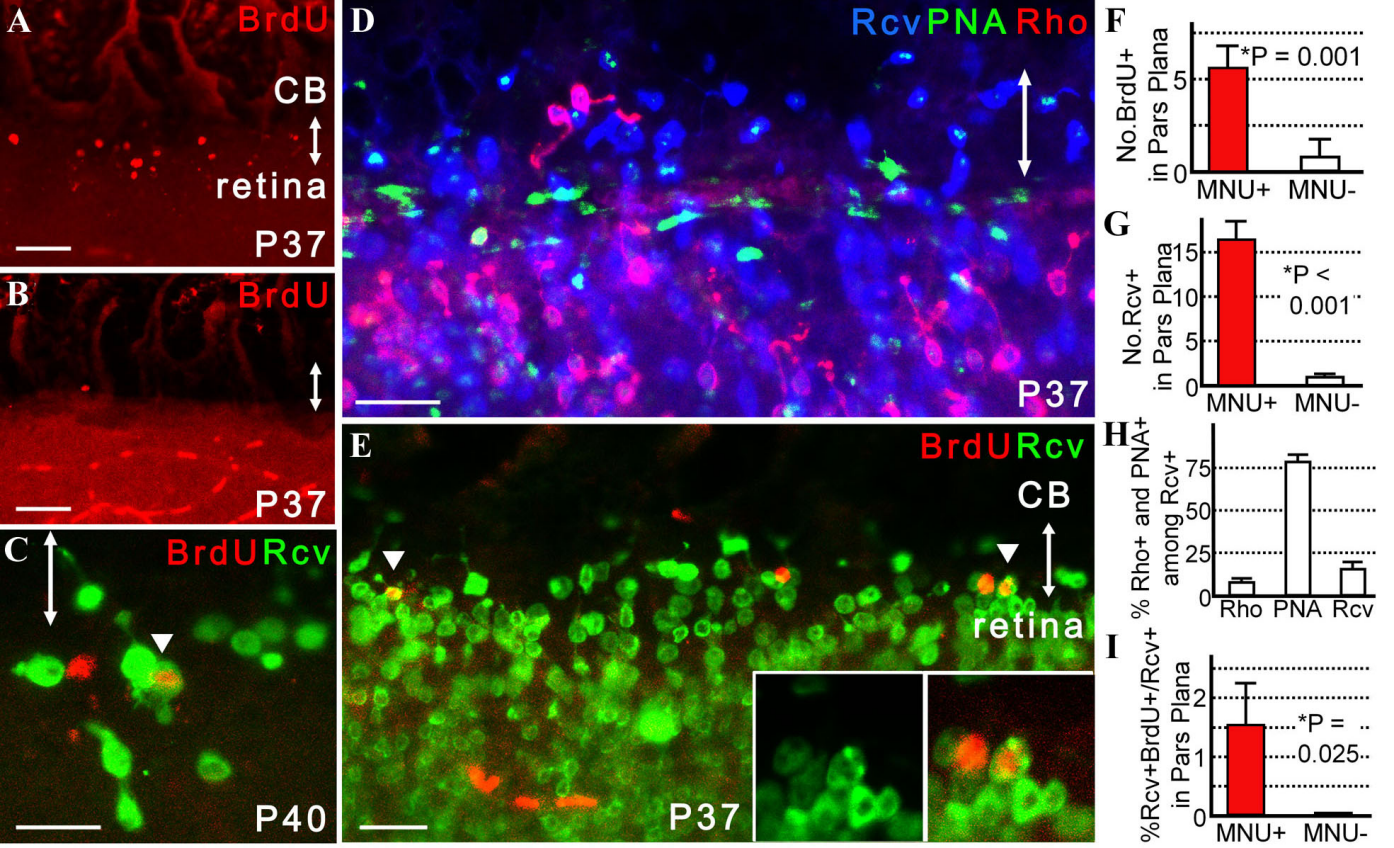
Neurogenesis in the pars plana in mice with MNU-induced photoreceptor degeneration. Double-headed arrows indicate the pars plana. MNU was injected at P30 and BrdU (50 mg/kg) was injected at P32. **A, B, F**: At P37, increased numbers of BrdU-positive cells were observed in the pars plana of MNU-treated mice (**A**) compared to untreated controls (**B**), which is summarized in **F**. Note that BrdU-positive cells in a linear alignment in the retina of **B** were blood cells in retinal vessels. **C:** Representative image of a cell positive for both recoverin and BrdU (arrowhead) in the peripheral retina at P40. **D,H**: Proportions of cells positive for PNA (cone) or rhodopsin (Rho; rod) among recoverin-positive cells in the pars plana (**H**) are presented. Note that the abrupt alteration in the cone-rod ratio signified the cilioretinal border. **E, I:** Rare cells positive for both recoverin and BrdU were identified in the pars plana of MNU-treated mice (**E**; n=8); such cells were never observed in control mice (**I**; n=12). **G**: At P37, increased numbers of recoverin-positive cells were observed in the pars plana of MNU-treated mice (n=8) as compared to the controls (n=12). For **H**, after the number of recoverin-positive cells within 320 µm width of the pars plan were determined from a single optical scan (10.0 μm thick), the number of those also positive for rhodopsin and PNA were determined from the same image. Three independent images randomly obtained from the same eye were analyzed to calculate the proportion of recoverin-positive cells that were also positive for rhodopsin (Rho) or PNA (PNA) or neither (Rcv) per animal. Average proportion (%; mean±SEM) of cells for each of the three categories were determined from 8 animals. For **F, G**, and **I,** the number of cells positive for BrdU, recoverin, or both within 320 µm width of the pars plana were determined from an optical scan (10.0 μm thick). Values from three independent images randomly obtained from a same eye were averaged. Data extracted from totals of 8 MNU-treated mice and 12 control mice were statistically processed and were presented as means±SEM **A** and **B** are thick scans merged from 2 scans (each scan was 10.0 μm thick) while **C** is presented as a thin scan (a single scan 3.9 μm thick). **D** and **E** are also thick scans but merged from 3 scans (each scan was 10.0 μm thick). Scale bar equals 25 μm in **C-E** and 50 μm in **A** and **B**. Abbreviation: recoverin (Rcv).

In *rd1* mice with inherited retinal degeneration, many recoverin-positive cells were found in the adult pars plana, a proportion of which were generated after retinal histogenesis. No evidence of neurogenesis was observed after P30 in *rd1* mice (data not shown) or P12 in wild-type mice. Therefore, the important question of whether the pars plana of uninjured adult wild-type mice has the potential to newly produce immature retinal neurons/retinal precursors remains unsolved. Previously, we reported a marked increase in number of recoverin-positive cells in the pars plana of adult mice when acquired retinal degeneration was induced by MNU [16]. However, whether any of these cells were generated from proliferating retinal progenitors remains unknown. To resolve this issue, we injected MNU into wild-type mice at P30 and labeled proliferating cells with BrdU at P32. Mice eyes were subsequently collected for histological analyses. When the eyes were analyzed at P37, the number of BrdU-positive cells in the pars plana was increased by an estimated 4.3-fold in the MNU-treated animals as compared to untreated controls (Figures 8A,B,F). The number of recoverin-positive cells was also increased by approximately 17.1 fold in the MNU-treated mice (Figure 8G). Similar to the case of *rd1* mice, most of recoverin-positive cells in the pars plana were cells of cone photoreceptor lineage positive for PNA (roughly 77%) and a minor proportion were cells of rod photoreceptor lineage positive for rhodopsin (approximately 15%; Figure 8D,H). This was in contrast with the peripheral retina, which had a much larger proportion of rod photoreceptors. Therefore, the cilioretinal border could often be recognized as an abrupt alteration of cone-rod ratio (Figure 8D). However, most importantly, a small number of recoverin-positive cells in the pars plana was labeled with BrdU (approximately 1.52%); these cells were newly generated at or after P32. Such cells were observed infrequently also in the peripheral retina (Figure 8C). Some appeared to be cells of cone photoreceptor lineage (data not shown). No evidence of neurogenesis was detected in control mice (Figure 8I) at or after P32.

## Discussion

Our previous study showed that the pars plana has the potential to produce immature retinal neurons/retinal precursors during ocular development and in adult eyes with retinal damage [16]. In the present study, we provided direct in situ evidence that neurogenesis from proliferating retinal progenitors occurs in the pars plana not only during but also after retinal histogenesis in mice with inherited retinal degeneration or even in adult mice with acquired retinal degeneration. Meanwhile, no evidence of neurogenesis was detected in age-matched wild-type controls. The photoreceptor precursors in the pars plana illustrated in this study lacked pigments in their cell body and were therefore interpreted to be in the non-pigmented epithelial layer of the ciliary body, while in vivo-proven multipotent retinal stem cells were derived from the pigmented ciliary epithelium [9,10]. Such discrepancy may merely represent difference in their location with stages of differentiation (i.e., retinal stem cells in the pigmented layer may differentiate to become retinal precursors in the non-pigmented layer) or may indicate the presence of two distinct population of cells.

While the presence of photoreceptor precursors in the pars plana of mice treated with MNU has been already reported [16], this study provides a novel in vivo observation that part of these cells differentiate from mitotic retinal progenitors even after retinal histogenesis. Although, our results also showed that only a minor proportion (an estimated 3%) of retinal precursors in the pars plana of P30 *rd1* mice were generated after histogenesis, this is probably an underestimate and the true proportion is unknown. We demonstrated the presence of proliferating cells in the pars plana that were not labeled with a single intraperitoneal injection of 50 mg/kg BrdU, which may be partly explained by the following possibilities. First, as BrdU is incorporated only during the S-phase of the cell cycle, a portion of the unlabeled cells were probably in other phases of the cell cycle at the time of injection. Indeed, a previous study showed that almost half of the postnatal proliferating rat retinal progenitors were not in S-phase [31]. Second, as approximately 300 mg/kg of BrdU administration is needed to efficiently label the dividing cells in the brain [32], 50 mg/kg of BrdU used in the present pulse-chase assay was probably insufficient to label all the cells in S-phase. Third, retinal progenitors and their progeny that underwent multiple divisions after BrdU incorporation may have contained only undetectable amounts of BrdU. Finally, the incorporation of BrdU may have exerted a negative influence on the survival or differentiation of retinal progenitors [30]. Meanwhile, it is also possible that a large proportion of immature retinal neurons/precursors in the pars plana identified in the eyes with retinal degeneration differentiate from post-mitotic cells or transdifferentiate from non-neural epithelial cells. A possibility that some BrdU labeling might represent cells with MNU-induced DNA damage that were undergoing DNA synthesis/repair, not necessarily DNA synthesis due to mitosis, may also be considered.

The significance of the observation that immature retinal neurons/precursors in the pars plana and retinal neurons in the peripheral retina shared morphological features that differed from those of the posterior retina is unknown. One of the more attractive possibilities is that these cells share the same origins, which suggests the posterior migration of immature retinal neurons/retinal precursors generated in the pars plana. The presence of many recoverin-positive cells that encompassed both the pars plana and retina and the decrease in the number of immature retinal neurons/retinal precursors in the pars plana of *rd1* mice with time were among the findings consistent with this speculation.

The proportion of cone lineage cells among recoverin-positive cells was much greater than that of rod lineage cells in the pars plana. This appears to be an important feature of immature photoreceptors/photoreceptor precursors in the pars plana, which is the opposite of the adjacent retina that contains much larger number of rod photoreceptors compared to cone photoreceptors. Nonetheless, the preferential differentiation of cone photoreceptors in the pars plana may be a favorable observation when application of these cells for cell replacement therapy is to be considered in the future.

The results of the present study suggest the presence of essential cues for the differentiation of retinal progenitors into immature retinal neurons/retinal precursors within the pars plana. In addition, the observation consistent with the presence of clusters of rapidly dividing cells only in the pars plana implies the potential role of this zone in the expansion of retinal stem/progenitor cells. However, the potential of the adult mammalian pars plana to generate retinal neurons may be limited even under retinal damage, as evidence of neurogenesis was mostly restricted to the pars plana. This is unlike the adult CMZ in lower vertebrates, which contains numerous dividing retinal progenitors capable of replacing a large number of neurons throughout the retina.

In response to retinal damage, retinal progenitors proliferate and differentiate into immature retinal neurons/retinal precursors in the pars plana even after retinal histogenesis. This suggests that the pars plana may serve as a potential source for photoreceptor replacement therapy for the treatment of retinal disease in postnatal mammals.

## References

[1] Moshiri A, Close J, Reh TA (2004). Retinal stem cells and regeneration.. Int J Dev Biol.

[2] Reh TA, Fischer AJ (2001). Stem cells in the vertebrate retina.. Brain Behav Evol.

[3] Hitchcock P, Ochocinska M, Sieh A, Otteson D (2004). Persistent and injury-induced neurogenesis in the vertebrate retina.. Prog Retin Eye Res.

[4] Taupin P, Gage FH (2002). Adult neurogenesis and neural stem cells of the central nervous system in mammals.. J Neurosci Res.

[5] Ghashghaei HT, Lai C, Anton ES (2007). Neuronal migration in the adult brain: are we there yet?. Nat Rev Neurosci.

[6] Luskin MB (1993). Restricted proliferation and migration of postnatally generated neurons derived from the forebrain subventricular zone.. Neuron.

[7] Doetsch F, Caille I, Lim DA, Garcia-Verdugo JM (1999). varez-Buylla A. Subventricular zone astrocytes are neural stem cells in the adult mammalian brain.. Cell.

[8] Li H, Liu H, Heller S (2003). Pluripotent stem cells from the adult mouse inner ear.. Nat Med.

[9] Tropepe V, Coles BL, Chiasson BJ, Horsford DJ, Elia AJ, McInnes RR (2000). van der KD. Retinal stem cells in the adult mammalian eye.. Science.

[10] Ahmad I, Tang L, Pham H (2000). Identification of neural progenitors in the adult mammalian eye.. Biochem Biophys Res Commun.

[11] Haruta M, Kosaka M, Kanegae Y, Saito I, Inoue T, Kageyama R, Nishida A, Honda Y, Takahashi M (2001). Induction of photoreceptor-specific phenotypes in adult mammalian iris tissue.. Nat Neurosci.

[12] Akagi T, Mandai M, Ooto S, Hirami Y, Osakada F, Kageyama R, Yoshimura N, Takahashi M (2004). Otx2 homeobox gene induces photoreceptor-specific phenotypes in cells derived from adult iris and ciliary tissue.. Invest Ophthalmol Vis Sci.

[13] Xu H, Sta Iglesia DD, Kielczewski JL, Valenta DF, Pease ME, Zack DJ, Quigley HA (2007). Characteristics of progenitor cells derived from adult ciliary body in mouse, rat, and human eyes.. Invest Ophthalmol Vis Sci.

[14] MacNeil A, Pearson RA, MacLaren RE, Smith AJ, Sowden JC, Ali RR (2007). Comparative analysis of progenitor cells isolated from the iris, pars plana, and ciliary body of the adult porcine eye.. Stem Cells.

[15] Coles BL, Angenieux B, Inoue T, Del Rio-Tsonis K, Spence JR, McInnes RR, Arsenijevic Y (2004). van der KD. Facile isolation and the characterization of human retinal stem cells.. Proc Natl Acad Sci USA.

[16] Nishiguchi KM, Kaneko H, Nakamura M, Kachi S, Terasaki H (2008). Identification of photoreceptor precursors in the pars plana during ocular development and after retinal injury.. Invest Ophthalmol Vis Sci.

[17] Jin K, Galvan V, Xie L, Mao XO, Gorostiza OF, Bredesen DE, Greenberg DA (2004). Enhanced neurogenesis in Alzheimer's disease transgenic (PDGF-APPSw,Ind) mice.. Proc Natl Acad Sci USA.

[18] Jin K, Peel AL, Mao XO, Xie L, Cottrell BA, Henshall DC, Greenberg DA (2004). Increased hippocampal neurogenesis in Alzheimer's disease.. Proc Natl Acad Sci USA.

[19] Felling RJ, Snyder MJ, Romanko MJ, Rothstein RP, Ziegler AN, Yang Z, Givogri MI, Bongarzone ER, Levison SW (2006). Neural stem/progenitor cells participate in the regenerative response to perinatal hypoxia/ischemia.. J Neurosci.

[20] Farber DB, Flannery JG, Bowes-Rickman C (1994). The rd mouse story: seventy years of research on an animal model of inherited retinal degeneration.. Prog Retin Eye Res.

[21] Nishiguchi KM, Nakamura M, Kaneko H, Kachi S, Terasaki H (2007). The role of VEGF and VEGFR2/Flk1 in proliferation of retinal progenitor cells in murine retinal degeneration.. Invest Ophthalmol Vis Sci.

[22] Scholzen T, Gerdes J (2000). The K_i_-67 protein: from the known and the unknown.. J Cell Physiol.

[23] Blanks JC, Johnson LV (1983). Selective lectin binding of the developing mouse retina.. J Comp Neurol.

[24] Haverkamp S, Grunert U, Wassle H (2001). The synaptic architecture of AMPA receptors at the cone pedicle of the primate retina.. J Neurosci.

[25] Yoshizawa K, Nambu H, Yang J, Oishi Y, Senzaki H, Shikata N, Miki H, Tsubura A (1999). Mechanisms of photoreceptor cell apoptosis induced by N-methyl-N-nitrosourea in Sprague-Dawley rats.. Lab Invest.

[26] Fei Y (2003). Development of the cone photoreceptor mosaic in the mouse retina revealed by fluorescent cones in transgenic mice.. Mol Vis.

[27] Olney JW (1968). An electron microscopic study of synapse formation, receptor outer segment development, and other aspects of developing mouse retina.. Invest Ophthalmol.

[28] Young RW (1985). Cell proliferation during postnatal development of the retina in the mouse.. Brain Res.

[29] Young RW (1985). Cell differentiation in the retina of the mouse.. Anat Rec.

[30] Sekerkova G, Ilijic E, Mugnaini E (2004). Bromodeoxyuridine administered during neurogenesis of the projection neurons causes cerebellar defects in rat.. J Comp Neurol.

[31] Alexiades MR, Cepko C (1996). Quantitative analysis of proliferation and cell cycle length during development of the rat retina.. Dev Dyn.

[32] Cameron HA, McKay RD (2001). Adult neurogenesis produces a large pool of new granule cells in the dentate gyrus.. J Comp Neurol.

